# A Rare Anatomical Variation of the Lesser Occipital Nerve

**DOI:** 10.7759/cureus.15901

**Published:** 2021-06-24

**Authors:** A. Bert Chabot, Joe Iwanaga, Aaron S Dumont, R. Shane Tubbs

**Affiliations:** 1 Department of Neurosurgery, Tulane University School of Medicine, New Orleans, USA; 2 Anatomical Sciences, St. George's University, St. George's, GRD; 3 Department of Neurosurgery and Structural & Cellular Biology, Tulane University School of Medicine, New Orleans, USA; 4 Neurosurgery and Ochsner Neuroscience Institute, Ochsner Health System, New Orleans, USA

**Keywords:** lesser occipital nerve, variation, cadaver, clinical anatomy, spinal accessory nerve

## Abstract

The lesser occipital nerve (LON) is a cutaneous branch of the cervical plexus that arises from the second and sometimes the third spinal nerve and innervates the scalp. During routine dissection of the neck, the LON was observed to arise directly from the spinal accessory nerve. The aberrant nerve measured 1.9 mm in diameter and 10.2 cm in length. Although anatomical variations of the LON such as duplication and triplication have been observed, we believe the origination of this nerve directly and exclusively from the spinal accessory nerve is exceedingly rare. The current case adds to the sparse literature on the variations of the LON and might be of interest to clinicians treating neurological conditions or surgeons operating in the area.

## Introduction

The lesser occipital nerve (LON) is a cutaneous branch of the cervical plexus and arises from the ventral rami of the second and sometimes the third cervical nerve [1,2]. It bends around the spinal accessory nerve, and then around the posterior margin of the sternocleidomastoid muscle to ascend parallel with its posterior edge [2]. The LON penetrates the deep cervical fascia near the skull base and ascends over the occipital region, where its auricular branch supplies sensory innervation to the upper and medial thirds of the auricular skin and posterior branches supply the adjacent scalp. It can have connections with the greater occipital nerve and great auricular nerves and, sometimes, the auricular branch of the facial nerve [1,2]. Between the external occipital protuberance and intermastoid line, it divides into its terminal medial and lateral branches [3]. While select variations in LON anatomy including duplication, triplication, passage through the “carefree” region of the posterior cervical triangle, and combined contributions from the spinal accessory nerve and C2 fibers have been described in the literature, cadaveric reports of the LON originating directly and exclusively from the spinal accessory nerve has, to our knowledge, not been described in the extant literature. Here, we discuss an extremely rare cadaveric case as well as review the literature for other reported variants of the LON.

## Case presentation

**Figure 1:**
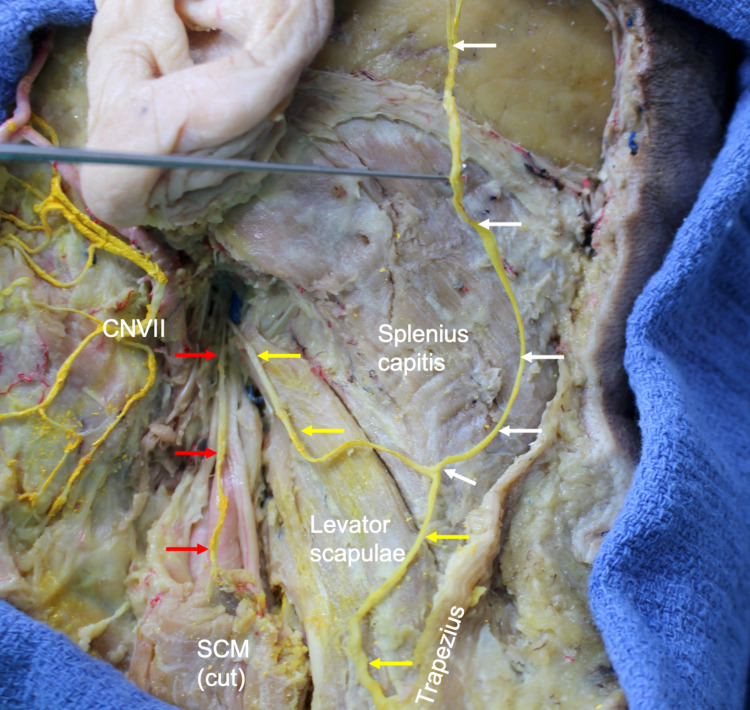
Dissection of the left neck in the case presented here. Note the two parts of the spinal accessory nerve, with an anterior branch (red arrows) traveling to the sternocleidomastoid, which has had its superior part removed, and a posterior branch (yellow arrows) traveling to the trapezius muscle. Note that superficial to the splenius capitis muscle, the posterior branch of the spinal accessory nerve gives rise to the LON (white arrows), which is lifted up with the dissecting probe. LON: lesser occipital nerve

**Figure 2:**
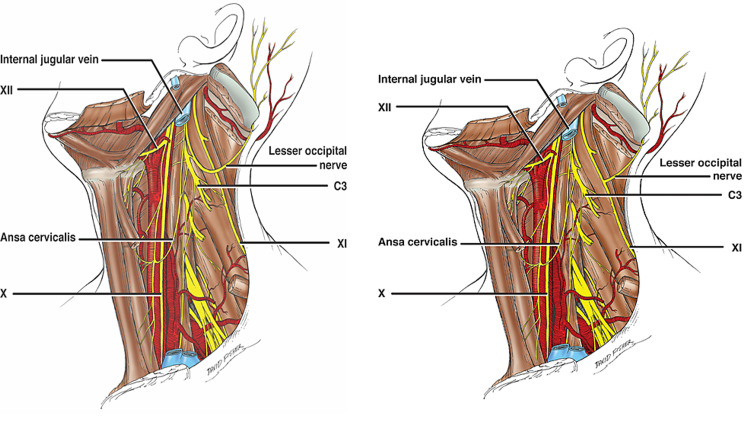
Schematic drawings indicating the normal LON (left) and the variant (right) presented here. LON: lesser occipital nerve

During routine anatomical dissection of the left neck of a 79-year-old at-death male cadaver, below the jugular foramen, the accessory nerve had two distinct branches that were more or less similar in diameter. The donor had died of natural causes. One branch traveled anteriorly and distally to supply the sternocleidomastoid muscle and the other traveled posteriorly and distally to innervate the trapezius muscle (Figure 1). The branch to the trapezius muscle, in the posterior cervical triangle, gave rise to an ascending branch that supplied the skin of the posterior auricle and scalp posterior to the ear. This was determined to be the LON and arose from the spinal accessory nerve superficial to the splenius capitis muscle (Figures 1, 2). The nerve was 1.9 mm in diameter and 10.2 cm in length. These measurements were made with a digital micrometer (Mitutoyo, Japan). No branches of the cervical plexus communicated with this nerve. Additionally, no cervical nerve branches were identified communicating with the sternocleidomastoid or trapezius muscles or the spinal accessory nerve. No additional nerve variations were noted in the areas dissected, and no gross evidence of pathology or previous surgery was found. The contralateral side of this cadaveric specimen was found to have a normal LON arising from the C2 spinal nerve without any connections to the spinal accessory nerve.

## Discussion

Duplication of the LON has been reported by Lucas et al. and Gupta et al. [4,5], while Madhavi and Holla reported a case in which the LON was triplicated bilaterally [6]. Another rare variation reported was the passage of the LON through the “carefree” region of the posterior triangle [7]. Rarely, the LON has been seen arising from the spinal accessory nerve, but these cases have also had C2 connections to the latter nerve and thus probably represent the C2 fibers of the LON “hitchhiking” along the accessory nerve [8]. The case presented here, however, showed no cervical nerve connections to the accessory nerve and was derived exclusively from this nerve (Figure 2).

Discrepancies noted in the distances between the LON and the midline have resulted in the lack of a distinct anatomical site for administering anesthetic nerve blockade [2]. This makes the treatment of occipitoparietal migraines or cervicogenic headaches difficult, which may be caused by LON stretching or compression [2]. Indeed, while nerve blocks comprising a small dose of local anesthetic combined with corticosteroids have proven efficacious in alleviating symptoms of occipital neuralgia and migraines, accurate determination of the optimal injection site is crucial in the effective blockade of the affected nerve [1]. The findings of this case as they pertain to the clinical implications of an anomalous LON can be strengthened by the further study of cadavers to assess for similar and alternate variations of the nerve, which can aid in establishing a range of anatomical variants.

In the present case, the question arises as to where are the sensory fibers within the variant LON connected proximally if there are no cervical nerve connections via the cervical plexus? A few possibilities might exist. One explanation is that microganglia along the spinal accessory nerve have been reported and might contain sensory neurons serving the LON if it arises directly from the spinal accessory nerve [9]. Another possibility is that a dorsal root ganglion of the first spinal nerve has been found to be associated with the spinal accessory nerve via direct connections [10]. Such connections could theoretically carry afferent information from the LON to the spinal cord. Finally, as the spinal portion of the spinal accessory nerve arise from the upper five to six spinal cord segments, these fibers, although thought of as being exclusively motor in nature, could potentially have connections to the spinal nerves of C2 or C3 spinal cord segments, which normally give rise to the LON. Taken together, as the craniocervical region is embryologically a complex transitional area, any of these mechanisms might be possible. The development of such a finding, based on a single case report, would be speculative.

We sincerely thank those who donated their bodies to science so that anatomical research could be performed. Results from such research can potentially increase mankind’s overall knowledge which can then improve patient care. Therefore, these donors and their families deserve our highest gratitude [11].

## Conclusions

Origination of the LON directly from the spinal accessory nerve, without contribution from the cervical plexus, appears to be an extremely rare finding and, to our knowledge, has not been reported previously. This case report contributes to the scant literature regarding anatomical variations of the LON and spinal accessory nerves. In such rare cases, injury to the spinal accessory nerve could not only result in dysfunction of the sternocleidomastoid and trapezius muscles but also a sensory deficit in the distribution of the LON.
